# Continuous smartwatch monitoring after atrial fibrillation ablation: feasibility of burden estimation and association with quality of life

**DOI:** 10.3389/fcvm.2025.1695891

**Published:** 2026-01-12

**Authors:** João G. Almeida, Duarte Dias, Rafael Silva-Teixeira, Mafalda Carrington, Paulo Fonseca, Marco Oliveira, Helena Gonçalves, João Primo, Ricardo Fontes-Carvalho, Luís Azevedo, Sérgio Barra, Juan Pablo Martínez, Rute Almeida

**Affiliations:** 1 Cardiology Department, Unidade Local de Saúde Gaia Espinho, Vila Nova de Gaia, Portugal; 2 RISE-HEALTH, Department of Community Medicine, Information and Health Decision Sciences, Faculty of Medicine, University of Porto, Porto, Portugal; 3 RISE-HEALTH, Department of Surgery and Physiology, Faculty of Medicine, University of Porto, Porto, Portugal; 4 Cardiology Department, Luz Arrábida Hospital, Vila Nova De Gaia, Portugal; 5 Aragon Institute of Engineering Research (I3A), IIS-Aragon, University of Zaragoza, Zaragoza, Spain; 6 Centro de Investigación Biomédica en Red—Biomedicina, Bioingeniería y Nanomedicina (CIBER-BBN), Zaragoza, Spain

**Keywords:** atrial fibrillation, digital health, patient-reported outcomes, quality-of-life, smartwatch, Withings

## Abstract

**Introduction:**

Continuous atrial fibrillation burden assessment is clinically relevant but often limited by the invasiveness of current tools. Wearables offer a non-invasive alternative, but evidence in the post-ablation setting is limited. We assessed the feasibility of smartwatch-based atrial fibrillation burden quantification after catheter ablation and its association with quality of life.

**Methods:**

In this prospective, single-centre study, patients undergoing atrial fibrillation ablation entered a 12-month digital follow-up program using a smartwatch (daily electrocardiogram recommended). Atrial fibrillation burden was defined as the percentage of monitored days with atrial fibrillation-detected electrocardiograms. A Bayesian multivariable model examined the association between atrial fibrillation burden and quality-of-life score (AFEQT).

**Results:**

Twenty patients (mean age 52.6 ± 10.3 years; 10% female) were enrolled. Over 12 months, 3,604 electrocardiograms were collected (mean 180 per participant); atrial fibrillation was detected in 55%. Electrocardiograms were submitted on 36% of days. Median atrial fibrillation burden was 1.4% (range: 0%–25%). AFEQT improved significantly over time (+1.71 points/month; 95% Credible Interval: 0.78–2.65), with 69% achieving a clinically meaningful improvement. Unlike atrial fibrillation recurrence, higher atrial fibrillation burden was associated with smaller AFEQT gains (interaction estimate: −0.23; 95% Credible Interval: −0.40 to −0.06). Each 1% increase in atrial fibrillation burden corresponded to an estimated 2.8-point lower AFEQT at 12 months.

**Conclusion:**

Smartwatch-based monitoring of atrial fibrillation burden is feasible after ablation. Higher atrial fibrillation burden was associated with reduced improvement in quality of life, supporting its value as a patient-centred outcome metric.

## Introduction

Atrial fibrillation (AF) is the most common sustained cardiac arrhythmia in adults, with a rising global prevalence (1). It is associated with substantial morbidity, including stroke, heart failure, cardiovascular death, and impaired quality of life (QoL), imposing significant healthcare costs (2). Early rhythm control therapy has been shown to improve outcomes (3), and catheter ablation is a well-established intervention that effectively reduces AF burden and significantly improves QoL in patients with symptomatic paroxysmal or persistent AF (4, 5).

Traditionally, ablation outcomes have been reported in binary terms (recurrence or no recurrence) based on intermittent monitoring. However, this approach may underestimate the overall impact of AF ablation (6). AF burden, defined as the proportion of time a patient is in AF, has emerged as a clinically meaningful metric, associated with symptoms, QoL, and adverse outcomes (7, 8). Recent studies suggest that a reduction in AF burden, rather than mere absence of recurrence, may better capture the true impact of ablation on outcomes (9). Accordingly, AF burden reduction has been proposed as a therapeutic target (10).

While implantable cardiac monitors remain the most widely accepted method for assessing continuous AF burden, their widespread adoption is limited by their invasive nature and cost (11). Wearable devices offer a non-invasive and scalable alternative (12). While promising, the feasibility and reliability of these tools for estimating post-ablation AF burden in real-world settings remain underexplored (13).

In this study, we evaluate the feasibility of quantifying AF burden using smartwatch-based monitoring in patients who underwent catheter ablation for AF. Additionally, we aim to assess adherence to monitoring and the impact of smartwatch-derived AF burden on QoL.

## Materials and methods

### Study design

This was a non-randomized single-center prospective observational study that included patients who underwent AF ablation at a Portuguese tertiary hospital.

A sample of patients was selected to use a commercially available smartwatch for 12 months after the procedure. Patients were eligible if they had paroxysmal or persistent AF, were scheduled for either first-time or repeat catheter ablation, were >18 years old, had a compatible smartphone, and were willing to use the smartwatch. Exclusion criteria included cardiac implantable devices, inability to operate the smartphone and/or smartwatch independently, and lack of internet access or mobile data required for mobile application synchronisation. The recruitment period extended from August 2022 to September 2023. Participants were selected consecutively based on the availability of devices and the research team's capacity, reflecting a convenience sampling approach. Of 23 patients invited to participate, 20 were enrolled. Three eligible participants declined enrollment, all of whom were female patients. Reasons for refusal included concerns about technology use (*n* = 1), physical discomfort with wearing a smartwatch (*n* = 1), and aesthetic concerns (*n* = 1). As our standard for post-AF ablation follow-up care, patients were enrolled in a digital follow-up program and invited to install the monitoring mobile application one week before the procedure (14). Clinical data were collected using a web platform (Promptly—Software Solutions for Health Measures, https://promptlyhealth.com).

This study was conducted in accordance with the ethical principles outlined in the Declaration of Helsinki. The Unidade Local de Saúde Gaia e Espinho's ethical committee approved the study protocol, and written informed consent was obtained for all patients.

### Procedure description

According to current guidelines, all patients received oral anticoagulation for at least four weeks before the ablation procedure and continued anticoagulation for at least two months post-procedure. On the day of ablation, cardiac computed tomography was performed to exclude left atrial thrombus. Pulmonary vein isolation was achieved using radiofrequency ablation (very high-power/short duration), second-generation cryoballoon, or pulsed-field ablation, with additional ablation performed at the operator's discretion. All procedures were conducted under general anaesthesia. Procedural success was defined as confirmed electrical isolation of all pulmonary veins. Additional procedure details are provided in the supplementary methods.

### Follow-up and outcomes

**Figure 1:**
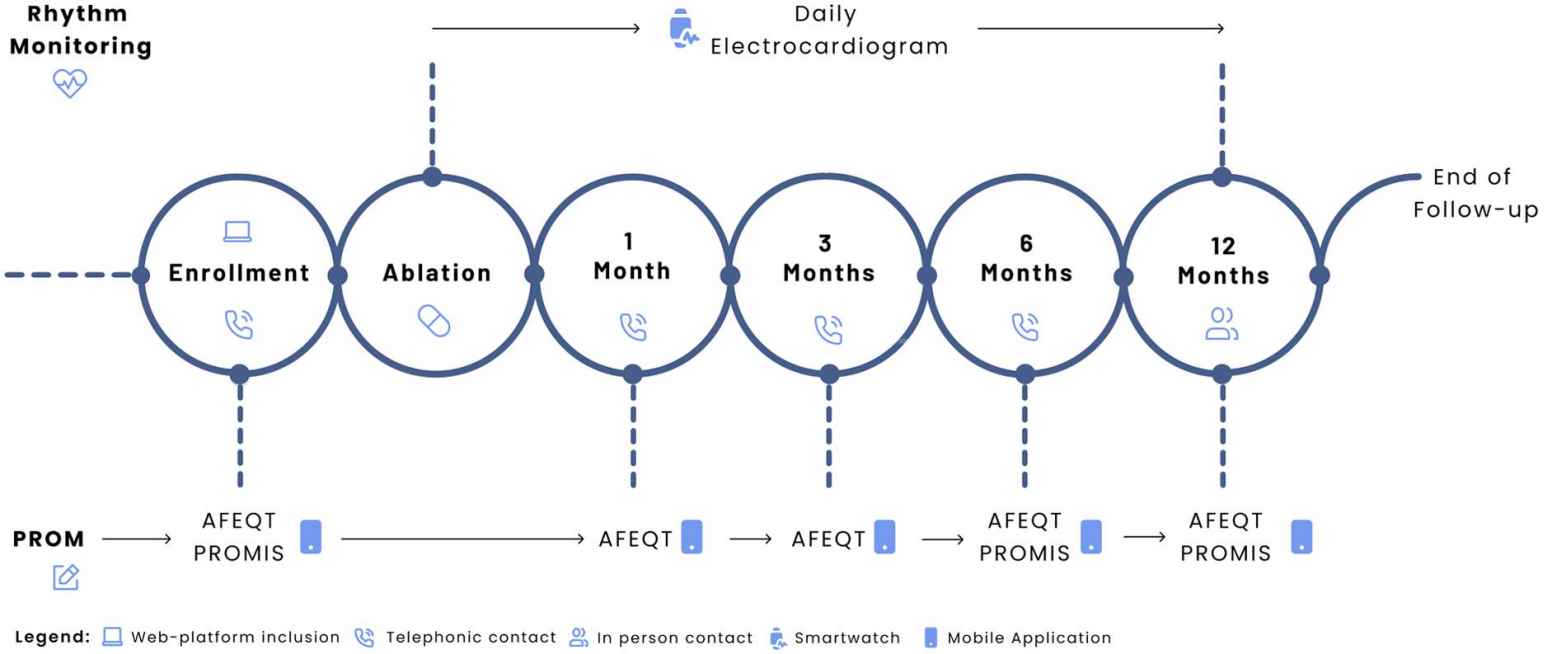
Study design. Adapted from “Digital follow-up workflow. AFEQT = Atrial Fibrillation Effect on Quality-of-Life; ECG = electrocardiogram; PROM = patient-reported outcome measure; PROMIS = Patient-Reported Outcomes Measurement Information System” by João G. Almeida, Rafael Teixeira, Inês Neves, Mafalda Carrington, Paulo Fonseca, Marco Oliveira, Helena Gonçalves, João Primo, Ricardo Fontes-Carvalho, Sérgio Barra, Juan Pablo Martínez, and Rute Almeida, licensed under CC BY-NC-ND.

A digital-blended care model, previously described by our group (14), was employed. It combines scheduled in-person visits, telephonic consultations, and remote monitoring via a digital health platform (Figure 1). This includes a mobile application for patients (Promptly v2.5.7, Promptly Health, 2024), which supports reporting symptoms and vital signs, as well as completing health-related questionnaires.

Patient-reported outcomes (PROMs) were assessed using the validated Portuguese versions of the Atrial Fibrillation Effect on Quality of Life (AFEQT) questionnaire and four Patient-Reported Outcomes Measurement Information System (PROMIS) short-form tools. The AFEQT instrument evaluates symptom burden, daily activities, and treatment concerns, with scores ranging from 0 to 100 (higher scores indicating better QoL) (15). A change of 5 points or more was considered clinically meaningful (16). Patients were prompted to complete the AFEQT via the mobile application at enrollment, and at one, three, six, and 12 months. PROMIS questionnaires addressed Cognitive Function (v2.0) (17), Physical Function (v2.0) (18), Anxiety (v1.0) (19), and Depression (v1.0) (19) using 4-item short forms. Each domain uses a standardised T-score, where a score of 50 represents the mean of a reference population; scores above or below 50 reflect better or worse function, respectively. PROMIS assessments were made at enrollment, six months, and 12 months. The completeness rate was calculated as the proportion of completed and scheduled questionnaires at each time point.

AF recurrence was defined as the detection of AF on at least one 30-second single-lead electrocardiogram (ECG), as classified by the smartwatch's automated algorithm, irrespective of timing after the procedure. For reporting purposes, early recurrence was defined as an event occurring within the first 8 weeks post-ablation (blanking period) (20), while late recurrence referred to events occurring thereafter. Antiarrhythmic intervention was defined as re-initiation of antiarrhythmic medication, repeat ablation, or electrical cardioversion.

### Smartwatch monitoring and AF burden estimation

After enrollment, patients received the smartwatch (Withings^®^ Scanwatch) in the first 24 h after the procedure. Beyond an automatic AF-detection algorithm based on the photoplethysmography (PPG) sensor, this wearable also allows recording a single-lead 30-second ECG. For the initial configuration, patients could choose to wear the watch on either arm. They received recommendations to ensure continuous skin contact, and an initial ECG was performed for demonstration purposes. The recommendation was to perform the ECG seated with the wrist positioned on the left knee (similar to lead I ECG). They were instructed to use the smartwatch daily, with as few interruptions as possible, and perform an ECG every day, when arrhythmia symptoms occurred or when recommended by the automatic AF- detection algorithm. This smartwatch records the PPG waveform every 10 min. This method provides a near-continuous rhythm monitoring by regularly checking the PPG waveform for signs of arrhythmia. After the first detection of an irregular rhythm, the smartwatch opens a 24-hour window. When 10 PPG checks meet the requirement for arrhythmia, the patient is recommended to perform an ECG to confirm AF. ECG classification was automatically performed by the previously validated Withings's algorithm as normal, AF, or inconclusive (21). All data was stored in the Withings’ Health Mate App and synced with the Promptly App.

Regarding daily ECG compliance, Frequency (%) was calculated as (number of days with at least a smartwatch ECG/365) × 100, representing the proportion of days each patient submitted a smartwatch-based ECG during the 12-month follow-up period. Additionally, Density was calculated as (number of smartwatch ECG measurements/365), which quantifies how frequently ECG measurements were performed daily, on average. This study was designed to reflect a real-world clinical scenario, and there was no supervision for the device usage compliance. Traditionally, AF burden is derived from continuous rhythm invasive monitoring (11); the AF burden definition used in this study was based on the daily presence of AF, as determined by Withings's automated ECG classification algorithm. It was calculated as the number of days with at least one ECG classified as AF divided by the total number of days with an ECG recording, expressed as a percentage.

### Statistical analysis

Continuous variables are presented as mean ± standard deviation (SD) or median ± interquartile range (IQR), as appropriate. Normality was assessed through visual inspection of histograms and Q-Q plots. Comparisons of continuous variables were performed using either the Student's *t*-test or the Mann–Whitney *U*-test, depending on the distribution. Categorical variables were compared using the chi-square test or Fisher's exact test, as appropriate. AFEQT and PROMIS scores were analysed using repeated-measures linear mixed-effects models with time as a fixed effect and random intercepts to account for individual baseline differences. Frequentist statistical tests used two-sided *p*-values, with a significance level of 0.05. Given the ability to handle small samples, a more intuitive inferential framework, and deviations from the frequentist linear mixed model assumptions, we adopted a multivariable Bayesian multilevel linear modelling approach to evaluate the impact of AF burden on the AFEQT score. Bayesian models were fitted with AFEQT score as the response variable, time, AF burden, and their interaction as fixed effects. Covariates were selected based on established associations with AF burden and QoL in prior ablation studies (8, 22–24): CHA₂DS₂-VASc score, baseline AFEQT score, AF type, and indexed left atrial volume. Random intercepts were included to account for baseline differences between participants. Two models were tested: one considering AF burden as a continuous variable and another treating it as a binary variable (presence or absence of AF recurrence). We modelled the AFEQT score using a Gaussian distribution to retain interpretability on the original scale (0–100). While this model does not strictly constrain predicted values within bounds, diagnostic checks revealed that most predictions fell within the valid range, and residuals behaved acceptably. All models used weakly informative priors. Models were estimated using four Markov Chain Monte Carlo chains, each with 5.000 iterations (1.000 warm-up iterations). Model convergence was assessed using R^ statistics (target R^ = 1.00) and effective sample sizes for both bulk and tail estimates. Markov Chain Monte Carlo diagnostics are presented in Supplementary Methods. A 95% credible interval of the posterior distribution that excluded zero was interpreted as providing evidence that the effect was consistent with the estimate. To compare model performance, we conducted leave-one-out cross-validation using the expected log predictive density (higher values suggest better predictive fit). Analysis was performed using R statistical software version 4.4.1 (The R Foundation for Statistical Computing, Vienna, Austria). Bayesian analysis was done using Stan (via the brms package in R, version 2.22.0).

## Results

### Baseline characteristics and clinical outcomes

Between August 2022 and September 2023, 20 patients were enrolled (mean age 52.6 ± 10.3 years, 10.0% female, 70% with paroxysmal AF) (Table 1). The median CHADSVASc score was 1, with a median time since AF diagnosis of 2 years. Pulmonary vein isolation was achieved in all cases, mainly with radiofrequency (75.0%). Additional cavotricuspid isthmus ablation was performed in two patients (10%) due to documented typical atrial flutter; no other additional ablation lesions were delivered. No procedural complications were observed. After the procedure, the median number of remote and in-person appointments per patient was 3 and 1, respectively. During the 12-month follow-up, nine patients (45%) had an early recurrence, and seven (35%) had a late recurrence. In total, 11 patients (55%) experienced at least one recurrence, and these patients were significantly older than the group without recurrence (56.9 vs. 47.4 years, *p* = 0.038), as shown in Table 1. An anti-arrhythmic intervention (electrical cardioversion, new anti-arrhythmic medication, or redo procedure) was needed in four patients, and one patient required an emergency department visit.

**Table 1:** Baseline characteristics of the study population, overall and stratified by atrial fibrillation recurrence status.

Variable	Overall (*n* = 20)	AT/AF recurrence		*P*-value
		No (*n* = 9)	Yes (*n* = 11)	
Age (years)	52.6 (10.3)	47.4 (12.2)	56.9 (6.3)	0.038*
Male	18 (90.0)	8 (88.9)	19 (90.9)	1.000
Body mass index (kg/m^2^)	26.6 (5.4)	25.6 (5.7)	29.1 (5.9)	0.325
Hypertension	9 (45.0)	3 (33.3)	6 (54.6)	0.406
Smoking (current/former)	4 (20.0)	2 (22.2)	2 (18.2)	1.000
Dyslipidemia	8 (40.0)	3 (33.3)	5 (45.5)	0.670
Sleep apnea	2 (10.0)	0 (0.0)	2 (18.2)	0.479
Prior stroke or transient ischemic attack	1 (5.0)	0 (0.0)	1 (9.1)	1.000
Type 2 diabetes mellitus	1 (5.0)	0 (0.0)	1 (9.1)	1.000
Coronary artery disease	1 (5.0)	1 (11.1)	0 (0.0)	0.450
Heart failure	2 (10.0)	2 (22.2)	0 (0.0)	0.190
CHA2DS2-VASc score	1.0 (1.0)	1.0 (1.0)	1.0 (1.0)	0.967
Systolic dysfunction	2 (10.5)	2 (25.0)	0 (0.0)	0.163
Moderate or severe valvular disease	1 (5.0)	1 (11.1)	0 (0.0)	0.450
Left atrial volume (mL/m^2^)	38.2 (12.4)	37.9 (14.2)	38.4 (11.5)	0.930
Continued use of antiarrhythmic drugs	11 (55.0)	5 (55.6)	6 (54.6)	1.000
Oral anticoagulation	18 (90.0)	7 (77.8)	11 (100.0)	0.190
Paroxysmal atrial fibrillation	14 (70.0)	8 (88.9)	6 (54.6)	0.157
Time since diagnosis (years)	2.0 (3.0)	1.5 (3.0)	3.0 (4.5)	0.395
Prior electrical cardioversion	6 (33.3)	2 (28.6)	4 (36.4)	1.000
Previous ablation procedure	3 (15.0)	2 (22.2)	1 (9.1)	0.566
Ablation energy				1.000
Radiofrequency	15 (75.0)	8 (88.9)	7 (63.6)	
Cryoablation	4 (20.0)	1 (11.1)	3 (27.3)	
Pulse-field ablation	1 (5.0)	0 (0.0)	1 (9.1)	

Values are shown as *n* (%), mean (SD), or median (IQR), where applicable.

NOAC, non-vitamin k oral anticoagulant.

* Statistically significant between-group differences.

### Health-related questionnaires

**Figure 2:**
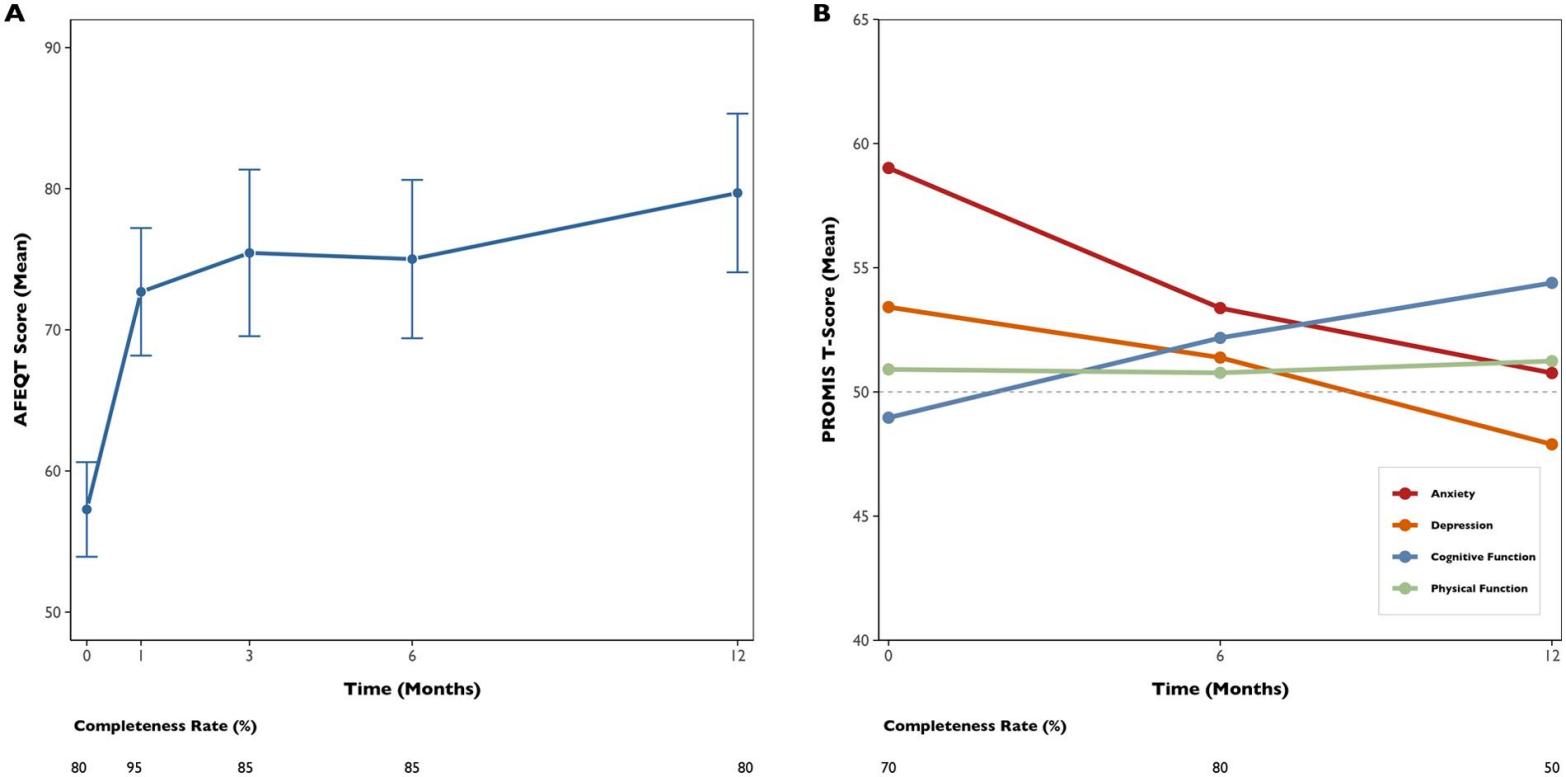
Trends in quality-of-life scores over 12 months following ablation. AFEQT mean score, with error bars, in the 12 months after ablation [left panel, **(A)**]. PROMIS mean T-Scores of the four domain questionnaires in the 12 months after ablation [right panel, **(B)**]. The round points represent specific time points of health-related questionnaire assessment.

During follow-up, symptoms were reported by six out of the 11 participants who had AF recurrence (54.5%), while five of these (45.5%) remained symptom-free despite experiencing a recurrence. A total of 85 AFEQT questionnaires were analysed. In total, 95% of participants completed at least two questionnaires, with a completeness rate of 80% (i.e., the proportion of completed questionnaires to those sent). The mean baseline AFEQT score (with lower scores indicating greater disability) was 57.3 points (SD 13.4). At 12 months, there was a significant improvement of +20.3 points [95% confidence interval (CI): 4.7–35.8, *p* = 0.015] (Figure 2A), with 69.2% of patients experiencing a clinically meaningful improvement in their AFEQT score. In a longitudinal model adjusting for repeated measures, the mean adjusted difference was +11.9 points (95% CI: 3.3–20.5, *p* = 0.008), as detailed in Table 2. Regarding PROMIS questionnaires, 160 assessments were analysed, with a completeness rate of 66.7%. Over time, there was a significant improvement in cognitive function (+5.2 points; 95% CI, 0.9–9.5; *p* = 0.020), alongside a significant reduction in depression scores (−5.7 points; 95% CI, −9.8 to −1.6; *p* = 0.009) and anxiety scores (−7.7 points; 95% CI, −13.0 to −2.4; *p* = 0.006) (Figure 2B). Although physical function showed a positive trend (+1.2 points; 95% CI, −2.8–5.2), this change was not statistically significant (*p* = 0.552).

**Table 2:** Estimated marginal means and mean adjusted differences of AFEQT and PROMIS scores between baseline and 12 months in the groups.

Quality-of-life questionnaires	Baseline	12-months	Mean adjusted difference (95% CI)	*p*-value
Mean overall AFEQT score (se)	66.2 (4.4)	78.1 (5.0)	+11.9 (3.3–20.5)	0.008*
Anxiety PROMIS mean t-score (se)	57.8 (2.4)	50.1 (2.6)	−7.7 (−13.0 to −2.4)	0.006*
Depression PROMIS mean t-score (se)	53.1 (2.0)	47.4 (2.2)	−5.7 (−9.8 to −1.6)	0.009*
Cognitive Function PROMIS mean t-score (se)	49.6 (2.1)	54.7 (2.2)	+5.2 (+0.9–9.5)	0.020*
Physical Function PROMIS mean t-score (se)	50.4 (1.6)	51.5 (1.8)	+1.2 (−2.8–5.2)	0.552

AFEQT, atrial fibrillation effect on quality-of-life; CI, confidence interval; PROMIS, patient-reported outcomes measurement information system; SE, standard error.

* Statistically significant differences.

### Smartwatch compliance and burden estimation

**Figure 3:**
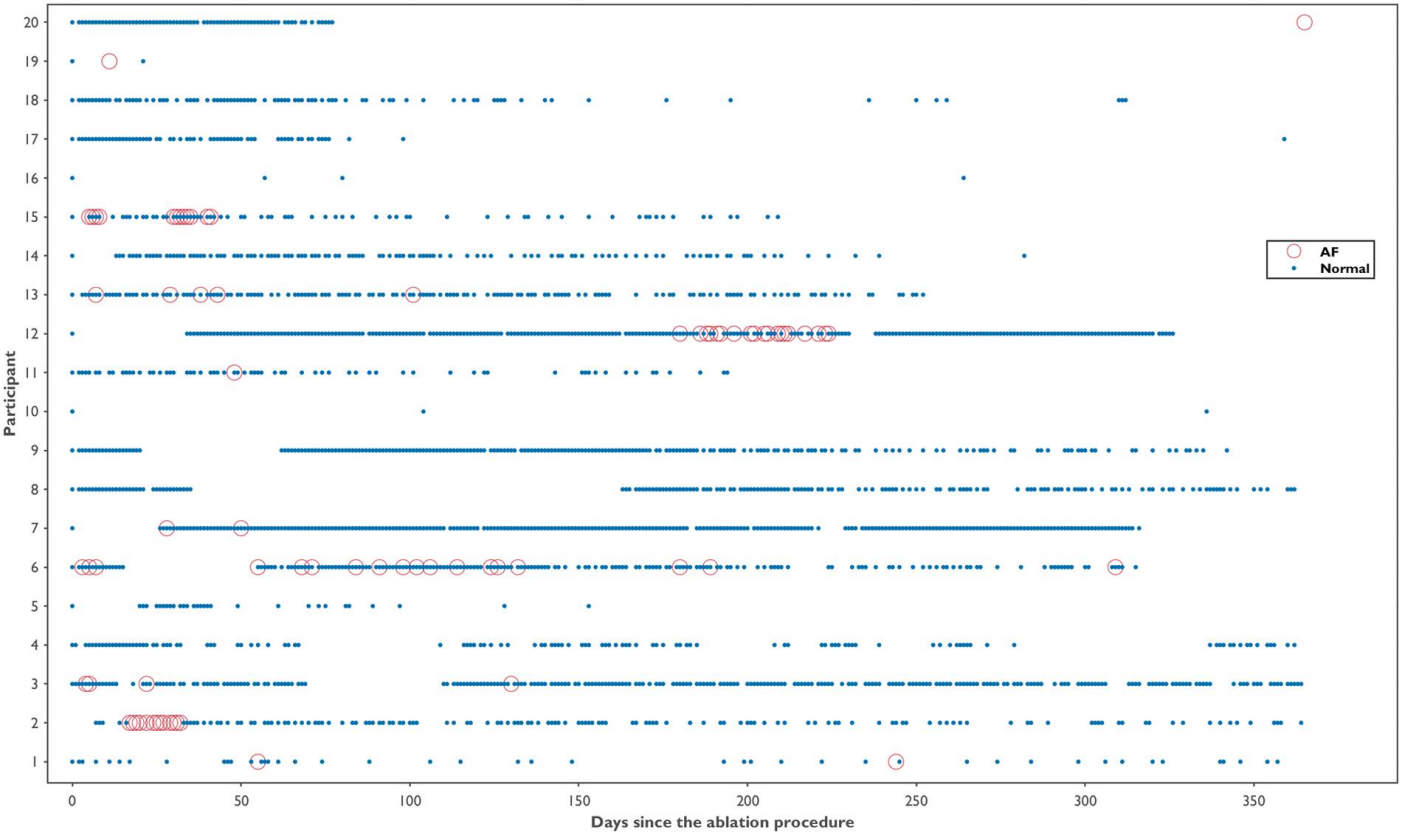
Temporal distribution of smartwatch ECG recordings by participant over 12 months post-ablation. Each blue dot represents a normal ECG recording; red circles indicate ECGs classified as atrial fibrillation. Gaps indicate days without ECG recordings.

**Figure 4:**
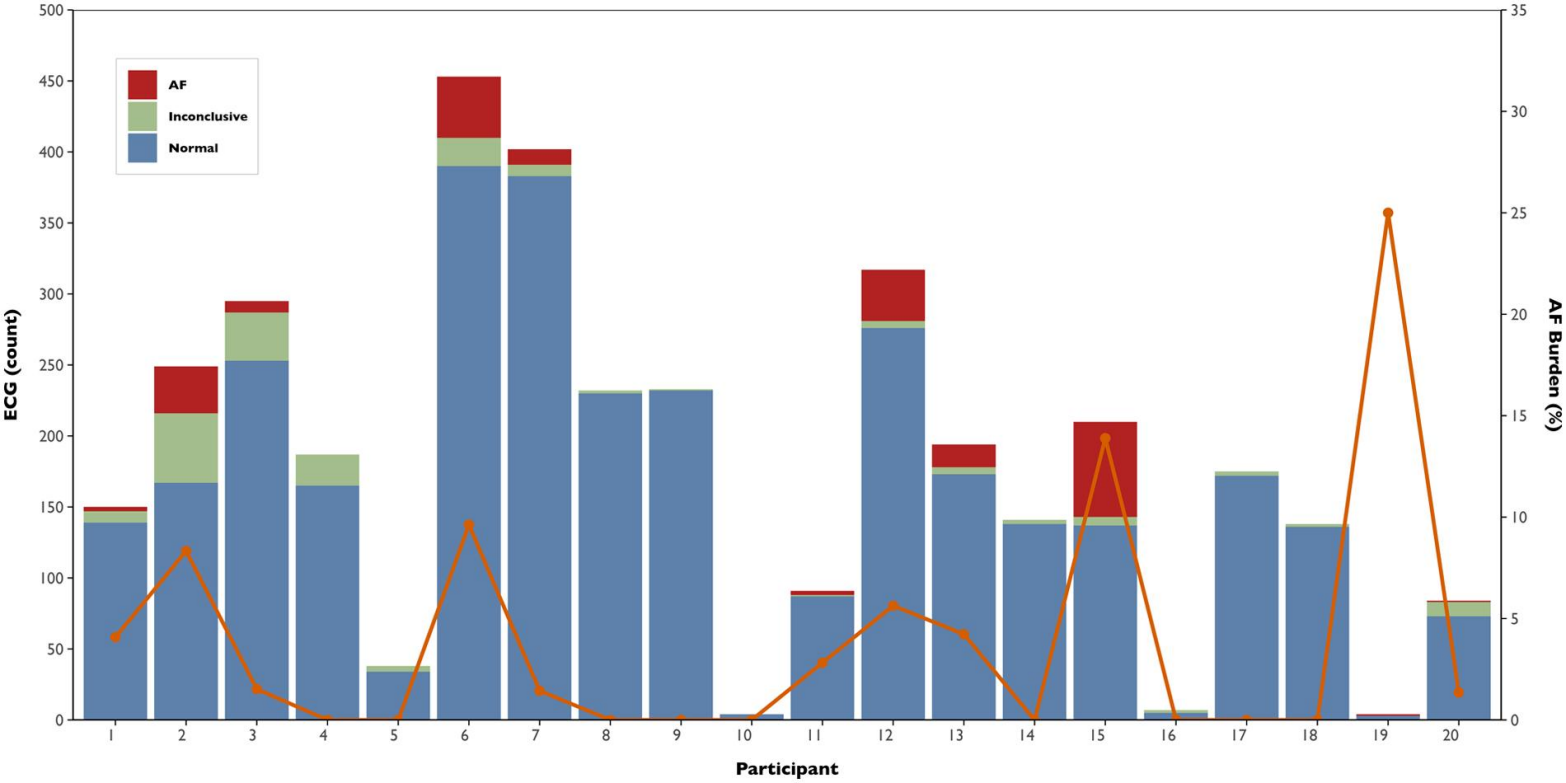
Atrial fibrillation burden and electrocardiogram classification by participant. Stacked bars represent the total number of smartwatch ECGs recorded per participant, classified as normal (blue), inconclusive (green), or AF (red). The orange line indicates the corresponding AF burden, defined as the percentage of monitored days with at least one AF-detected ECG.

Across all participants, 3,604 ECGs were collected over 12 months (mean per participant: 180 ± 126, range 4–453). On average, participants submitted ECGs on 35.8% of days (frequency), with a mean density of 0.5 ECG/day. The temporal distribution of the ECG recordings is displayed in Figure 3. A total of 185 inconclusive ECGs were recorded, with a median of 2.4% (IQR: 1.4%–10.8%) and 4.1% (IQR: 1.3%–7.4%) inconclusive ECGs per participant and day, respectively. The burden and number of ECGs collected per participant, along with their respective classifications, are shown in Figure 4. A total of 222 AF ECG recordings were collected. The median number of AF ECGs per participant was 1 (IQR: 0–12.3). Notably, nine participants (45%) had no AF episodes recorded, while the maximum number of AF ECGs in a single participant was 67. The AF burden, defined as the percentage of monitored days with one AF ECG, had a median of 1.4% (IQR: 0%–4.6%), ranging from 0% to 25%. Nineteen patients underwent 24-hour Holter monitoring during follow-up, with AF detected in only two cases. In both, the daily burden of AF would correspond to 100%. However, the actual time spent in AF during the recordings differed markedly: participant 6 spent 0.12% of the Holter in AF, while participant 20 spent 100%. In contrast, their smartwatch-derived AF burden over 12 months was 9.6% and 1.4%, respectively.

### Association between AF burden and AFEQT score

**Figure 5:**
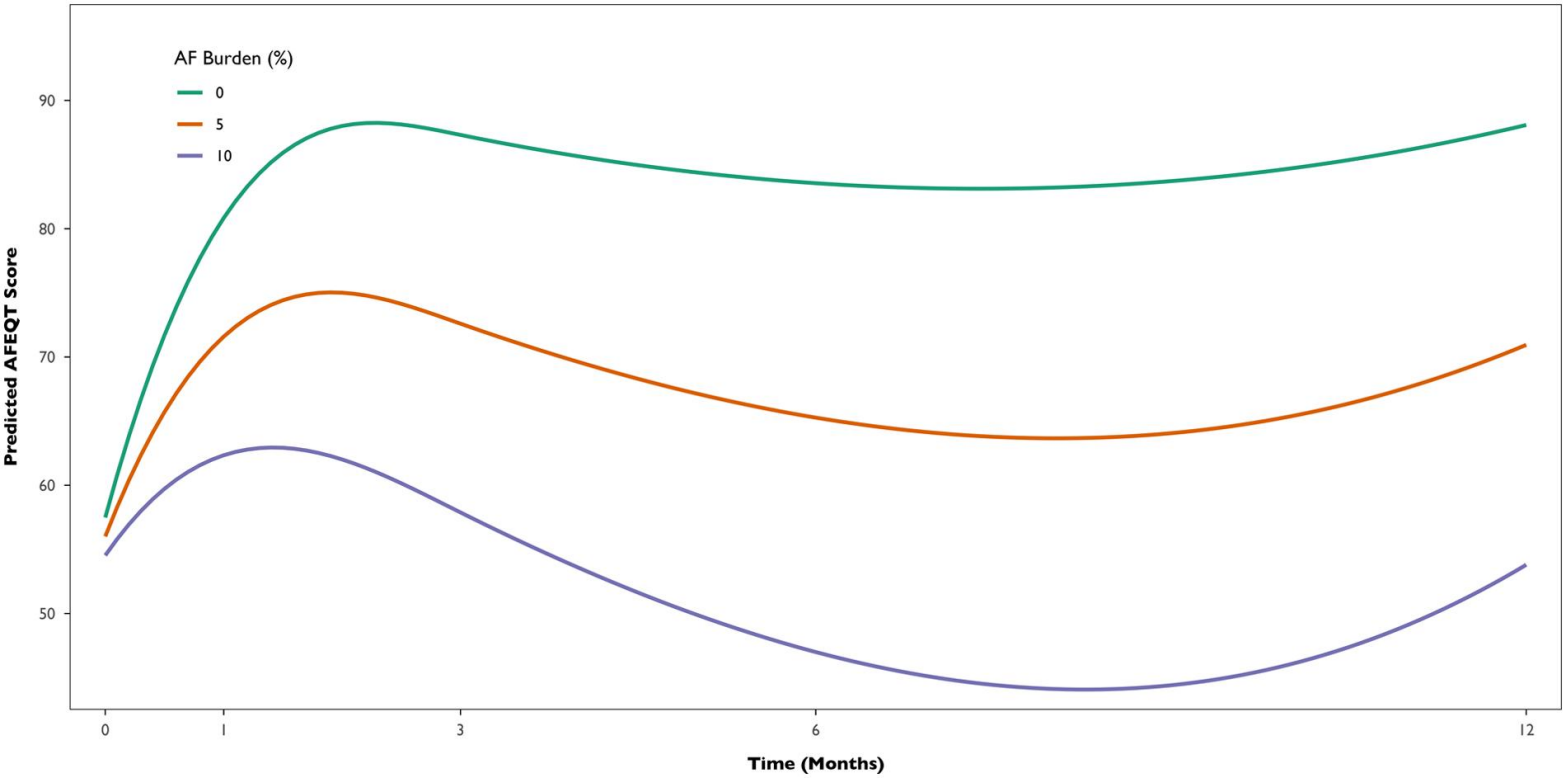
Predicted AFEQT score trajectories over 12 months by AF burden level (0%, 5%, and 10%). Model-based estimates derived from the Bayesian multilevel analysis with flexible time trajectories (splines), adjusted for CHA₂DS₂-VASc score, baseline AFEQT, AF type, and left atrial volume.

To evaluate the association between AF burden and QoL, we fitted a multivariable Bayesian multilevel linear model with AFEQT score as the outcome, and time, AF burden, and their interaction, CHADSVASc score, baseline AFEQT, AF type, and LA volume as fixed effects, including random intercepts for each participant. In the overall cohort, AFEQT score significantly improved over time after the procedure (Estimate for time = +1.71 points per month, 95% Credible Interval: 0.78–2.65). However, AF burden negatively impacted this trajectory. An interaction between time and AF burden was observed (Estimate = −0.23, 95% Credible Interval: −0.40 to −0.06), indicating that higher AF burden was associated with a smaller improvement in AFEQT scores over time. In contrast, no clear interaction was observed between time and AF recurrence in the binary model (Estimate = −0.67; 95% Credible Interval: −2.36–1.00), providing limited evidence that the presence or absence of recurrence alone was associated with changes in AFEQT scores over time. Additionally, model comparison using leave-one-out cross-validation indicated that the continuous burden model provided a better predictive fit, as reflected by a meaningful difference (>4) in expected log predictive density (−291 vs. −299). This supports more substantial evidence for a dose-response relationship between AF burden and AFEQT score. On average, each 1% increase in AF burden is associated with an estimated 2.8-point decrease in AFEQT score at 12 months. Figure 5 illustrates the multivariable model-predicted trajectories of AFEQT scores over 12 months, according to AF burden levels (0%, 5%, and 10%).

## Discussion

This prospective study demonstrated the feasibility of using a commercially available smartwatch to estimate AF burden in the first year after the ablation procedure. Over a 12-month period, patients submitted a substantial number of ECGs, enabling significant rhythm surveillance without the need for invasive monitoring. AF burden varied across participants and was quantifiable in real-world conditions. A higher AF burden was associated with smaller improvements in QoL, suggesting its potential value as a meaningful post-ablation outcome metric (Central Illustration).

### Clinical outcomes and quality of life

At 12 months, 55% of patients had documented recurrence, although nearly half were asymptomatic, consistent with contemporary trials with continuous rhythm monitoring (25). For example, in the CIRCA-DOSE trial, Andrade et al. compared cryoballoon and radiofrequency ablation in patients with paroxysmal AF using implantable cardiac monitors, reporting 1-year recurrence rates of approximately 53% (6). Notably, despite the high proportion of patients with recurrence in both studies, the actual AF burden remained low. In CIRCA-DOSE, the median AF burden at 12 months was 0%; in our cohort, it was similarly low, with a median of 1.4%. This highlights that recurrence does not necessarily indicate a high arrhythmic load, particularly when detected by continuous or near-continuous monitoring. The assessment of QoL after AF ablation is increasingly recognised as a key outcome in clinical practice and research. In our study, we achieved real-world high completeness rates of health-related questionnaires (67%–80%), comparable to major clinical trials (26, 27), supporting the feasibility and engagement of electronic PROM collection in this population. The magnitude of AFEQT score improvement (approximately 20 points) is also aligned with randomized trials, such as the recent sham-control AF ablation trial, SHAM-PVI (5). Furthermore, improvements in other domains of quality of life, including mental health (depression and anxiety) and cognitive function, were also observed in our study with PROMIS questionnaire assessments.

### Smartwatch compliance and burden estimation

Even though implantable cardiac monitors are the gold standard for rhythm monitoring after ablation in clinical trials, in clinical practice, post-ablation follow-up in routine clinical practice relies predominantly on short-duration continuous monitoring (e.g., 24 or 48-hour Holter), which almost invariably underestimates AF recurrence and overestimates the AF burden in patients with recurrences (28). This was reflected in our cohort, where short-term Holter monitoring failed to detect AF in 82% of cases, and in the remaining cases, showed markedly discrepant burden values compared to long-term smartwatch-derived estimates. Adherence to smartwatch use in our cohort was reasonable, with ECGs submitted on 36% of follow-up days. While adherence in our study was lower than in other non-invasive rhythm monitoring studies, such as TeleCheck-AF (29), this likely reflects the unsupervised, real-world conditions and the extended duration of follow-up. The multicentre TeleCheck-AF project was based on a 7-day monitoring with three daily PPG recordings, without ECG confirmation. In the sub-analysis of 98 patients from the DECAAF II trial (8), who submitted handheld ECG strips before and after the ablation, the proportion of days with submitted ECGs reached 67%. This increased adherence likely reflects the selection of a subpopulation that adhered to the pre-ablation ECG submission requirement (at least 10 strips) and the clinical trial context. While continuous rhythm monitoring remains the most accurate method for assessing arrhythmia burden, intermittent smartphone-based monitoring with one to three recordings per day has been shown to provide a reasonable estimate of AF burden, particularly over extended follow-up periods (28, 30, 31). When monitored continuously using implantable devices, patients with paroxysmal AF exhibit an average AF burden of around 11% (10), which can be reduced to less than 1% after the ablation procedure (6). In our study, we used a burden definition similar to that implemented in similar studies, like the DECAAF II (SMURDEN), which is based on the daily presence of AF assessed with a daily smartwatch ECG, reaching a median burden approximation of 1.4%. This value aligns with the mentioned studies with invasive continuous monitoring after ablation, although results should be interpreted carefully because of different population characteristics.

### Association between AF burden and QoL

AF burden is increasingly recognised as a clinically meaningful metric, with higher burden associated with greater symptom severity, impaired QoL, and increased risk of stroke and heart failure (10). Furthermore, consistent evidence links AF burden to reduced QoL in affected patients (32), while the evidence regarding the association with a single recurrence is more questionable (33). For instance, in a substudy of STAR-AF, QoL improved after ablation regardless of AF recurrence, unless AF burden (measured with Holter, ECG, or external loop recording) was high (27). The CAPTAF (34) and CIRCA-DOSE (35) trials, which utilized implantable cardiac monitors to assess AF burden, demonstrated an inverse relationship between burden and QoL, despite similar rates of 12-month recurrence. A key finding of our study is the inverse relationship between wearable-estimated AF burden and QoL improvement. In our Bayesian model, each 1% increase in AF burden was associated with a 2.8-point decrease in AFEQT at 12 months, while the association with 12-month recurrence was not observed. Even though the AF burden is highly dependent on the monitoring method, and the mentioned studies used different populations, monitoring times, and QoL questionnaires, the association between burden and QoL seems consistent. Our findings expand the growing body of evidence and suggest that assessing AF burden using a smartwatch could offer a pragmatic and scalable method for capturing patient-centered outcomes in clinical practice.

### Future directions

The last decade has seen exponential growth in the use of smart wearable devices for arrhythmia monitoring, particularly AF. There is a strong consensus that validated wearable devices utilizing PPG/ECG-based signals may provide a suitable method for assessing AF burden (32). A recent meta-analysis (36) has demonstrated that wearable devices can quantify AF burden with a mean error of approximately 1% compared to ECG monitoring. Despite this, wearable devices lack standardized AF analysis criteria and have several limitations, particularly false-positive rates (due to noise, movement artifacts, or ectopic beats), as well as digital literacy and socioeconomic barriers that may exclude vulnerable populations. While our results were encouraging, this pilot study is limited by its small convenience sample. Building on our findings, we plan to conduct a multicenter study with a larger and more diverse cohort to validate this strategy. Additional studies will be needed to validate non-invasive strategies against the gold standard and to determine whether wearable-guided clinical management leads to improvements in clinical outcomes.

### Limitations

This study has several limitations, including its single-center design and modest sample size, which limit external generalizability. However, the effective sample size for statistical inference is substantially higher due to the longitudinal data collection (3.604 ECGs and 85 AFEQT assessments), which, in association with Bayesian methods, enabled robust modeling. Although we adjusted for established confounders in our multivariable model, unmeasured confounding cannot be ruled out. Study recruitment was based on a convenience sample of patients who were able and willing to use a digital health platform and a smartwatch, introducing potential selection bias. Female patients are substantially underrepresented in our cohort, limiting our ability to assess sex-specific differences. This imbalance reflects the systematic sex disparities in AF ablation referral and research (37), and should be addressed in future studies through a sex-balanced recruitment. The 12-month follow-up duration does not capture late AF recurrences and their potential long-term impact on QoL. Extended follow-up studies are necessary to assess the durability of these associations over a longer period and to determine whether this strategy maintains accuracy and adherence beyond the first year. AF burden was estimated using intermittent, patient-initiated smartwatch ECG recordings rather than continuous rhythm monitoring. Gaps in ECG submissions and the episodic nature of AF can result in underestimation when no AF is detected, and overestimation when short symptomatic or smartwatch-detected AF episodes are recorded disproportionately. The absence of a comparator group with the gold standard (implantable loop recorder) also limits the evaluation of the clinical effectiveness of smartwatch-based AF burden monitoring. Additionally, while a prior validation study (21) demonstrated excellent sensitivity and specificity of the smartwatch's AF classification algorithm, clinicians did not independently review the ECGs.

## Conclusions

This prospective study demonstrated the feasibility of using a commercially available smartwatch to estimate AF burden in the first year following catheter ablation. Smartwatch-based monitoring enabled rhythm surveillance after the procedure, with sufficient adherence to estimate the burden in real-world conditions. Higher AF burden was associated with smaller improvements in quality of life, reinforcing its value as a clinically meaningful metric beyond simple recurrence. These findings support the use of wearable technology as a pragmatic and scalable tool for rhythm monitoring and patient-centred outcome assessment after AF ablation.

## Data Availability

The raw data supporting the conclusions of this article will be made available by the authors, without undue reservation.
