# Supplementary material for: Continuous smartwatch monitoring after atrial fibrillation ablation: feasibility of burden estimation and association with quality of life
Source: Front Cardiovasc Med. 2026 Jan 12;12:1695891. doi: 10.3389/fcvm.2025.1695891 (PMC12833617; doi:10.3389/fcvm.2025.1695891)
Supplement: Supplementary file 1 [file Datasheet1.pdf]

SUPPLEMENTARY MATERIAL

SUPPLEMENTARY METHODS

Ablation procedure

Radiofrequency Ablation (75% of cases):

Procedures were performed using a very high-power, short-duration (vHPSD) protocol. A 3D electroanatomic mapping system (CARTO 3®, Biosense Webster) was utilized for all cases. Left atrial geometry and voltage mapping were created using a high-density multipolar mapping catheter (PentaRay®, Biosense Webster). Point-by-point pulmonary vein isolation was performed using an irrigated contact force-sensing catheter (Qdot®, Biosense Webster) with power delivery of 90W for 4 seconds (vHPSD). A deflectable sheath (Vizigo®, Biosense Webster) was used in all cases. Successful isolation was confirmed by remapping, demonstrating the absence of signals within the pulmonary veins (entrance block).

Cryoballoon Ablation (20% of cases):

Cryoablation was performed using the second-generation 28-mm cryoballoon catheter (Arctic Front Advance®, Medtronic). Pulmonary vein occlusion was verified with contrast injection. Energy delivery consisted of a single freeze of 240 seconds per vein. Phrenic nerve pacing was performed during right-sided ablation to monitor diaphragmatic function. Procedural success was verified by the absence of pulmonary vein potentials recorded by the circular mapping catheter.

Pulsed-Field Ablation (5% of cases):

One procedure was performed using the FARAPULSE® Pulsed Field Ablation System (Boston Scientific). Similar to the radiofrequency workflow, a 3D electroanatomic map was created using a high-density multipolar mapping catheter (PentaRay®, Biosense Webster). Energy was delivered using the FARAWAVE® ablation catheter, with eight applications per vein (four in the "basket" configuration and four in the "flower" configuration). Catheter rotation was performed between applications to ensure circumferential coverage. Successful ablation was confirmed by remapping, demonstrating the absence of signals within the pulmonary veins (entrance block).

Bayesian multilevel linear modelling

All analyses used the default weakly informative priors provided by brms :

- Regression coefficients (fixed effects): Flat (uniform) priors for all predictors (AF burden, time, their interaction, CHA_2DS_2-VASc, baseline AFEQT, AF type, and indexed left atrial volume).
- Intercept: Student's t-distribution with 3 degrees of freedom, centered near the sample mean of the outcome (student_t(3, 75.9, 24.7)).
- Standard deviations of random effects (intercepts for participant ID): Student's t-distribution with 3 degrees of freedom, mean 0, scale 24.7 (student_t(3, 0, 24.7)), constrained to be positive (half-t).
- Residual standard deviation (sigma): Student's t-distribution with 3 degrees of freedom, mean 0, scale 24.7 (student_t(3, 0, 24.7)), constrained to be positive (half-t).

Bayesian model diagnostics:

Convergence was evaluated by inspecting autocorrelation plots (Supplementary Figure 1 and 2), $\hat{R}$ statistics, and effective sample sizes (Bulk_ESS and Tail_ESS) (Supplementary Table 1 and 2). All $\hat{R}$ values were close to 1.00, and effective sample sizes were satisfactory, indicating good chain mixing and convergence (Supplementary Figure 3 and 4). Model fit was assessed through several posterior predictive checks (PPC). Visual inspection of density overlays (Supplementary Figure 5 and 6) confirmed that the models captured the observed data distribution appropriately.

Supplementary Figure 1. Autocorrelation plots of Markov Chain Monte Carlo (MCMC) samples for model parameters of the continuous burden model.

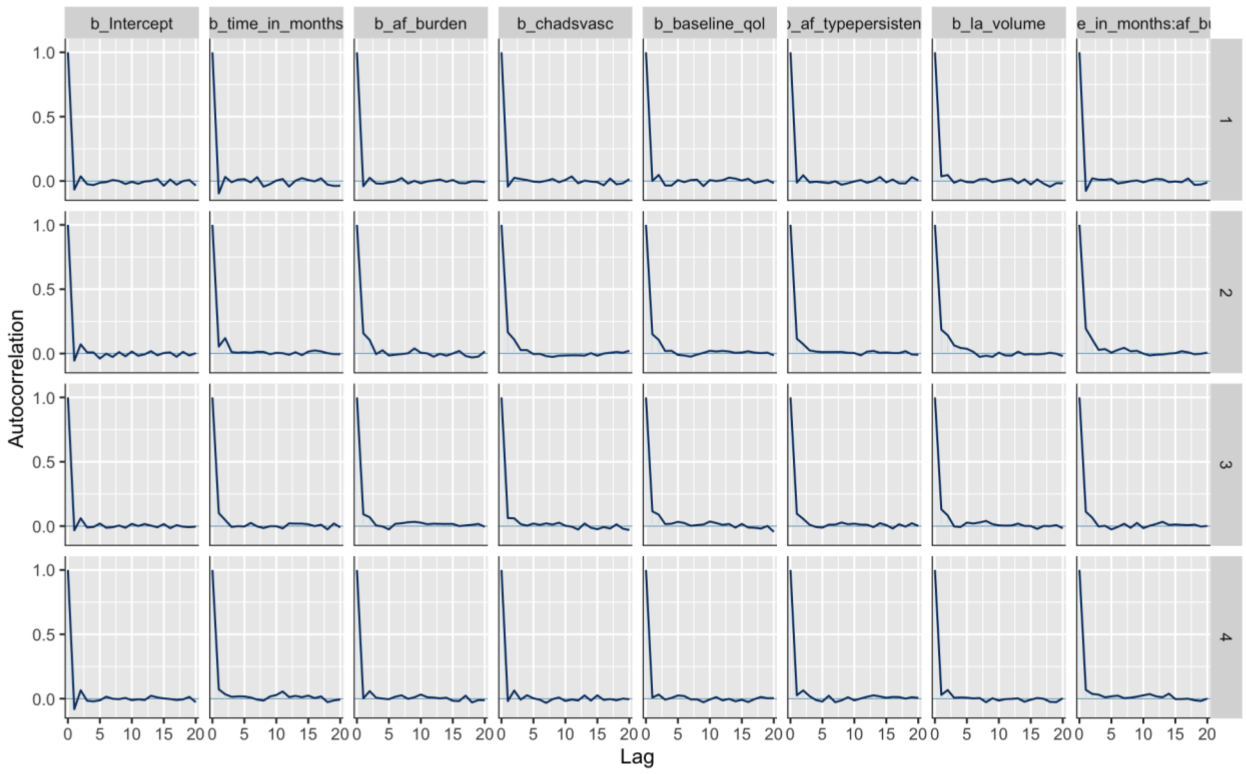

Supplementary Figure 2. Autocorrelation plots of MCMC samples for model parameters of the binary burden model.

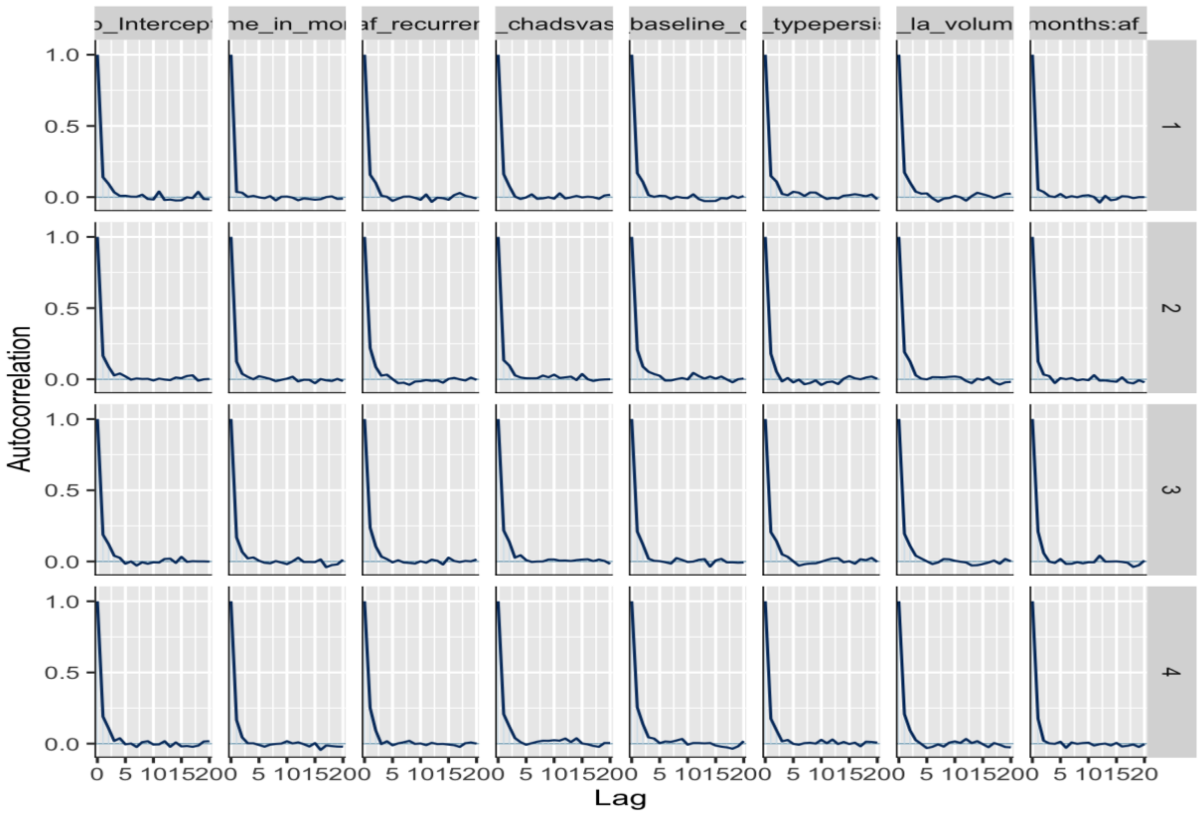

Supplementary Figure 3. Trace and posterior density plots for continuous burden model. Each row shows the posterior distribution (left) and trace plot across MCMC iterations (right) for the respective model parameter. Good chain mixing and overlapping density curves support adequate convergence.

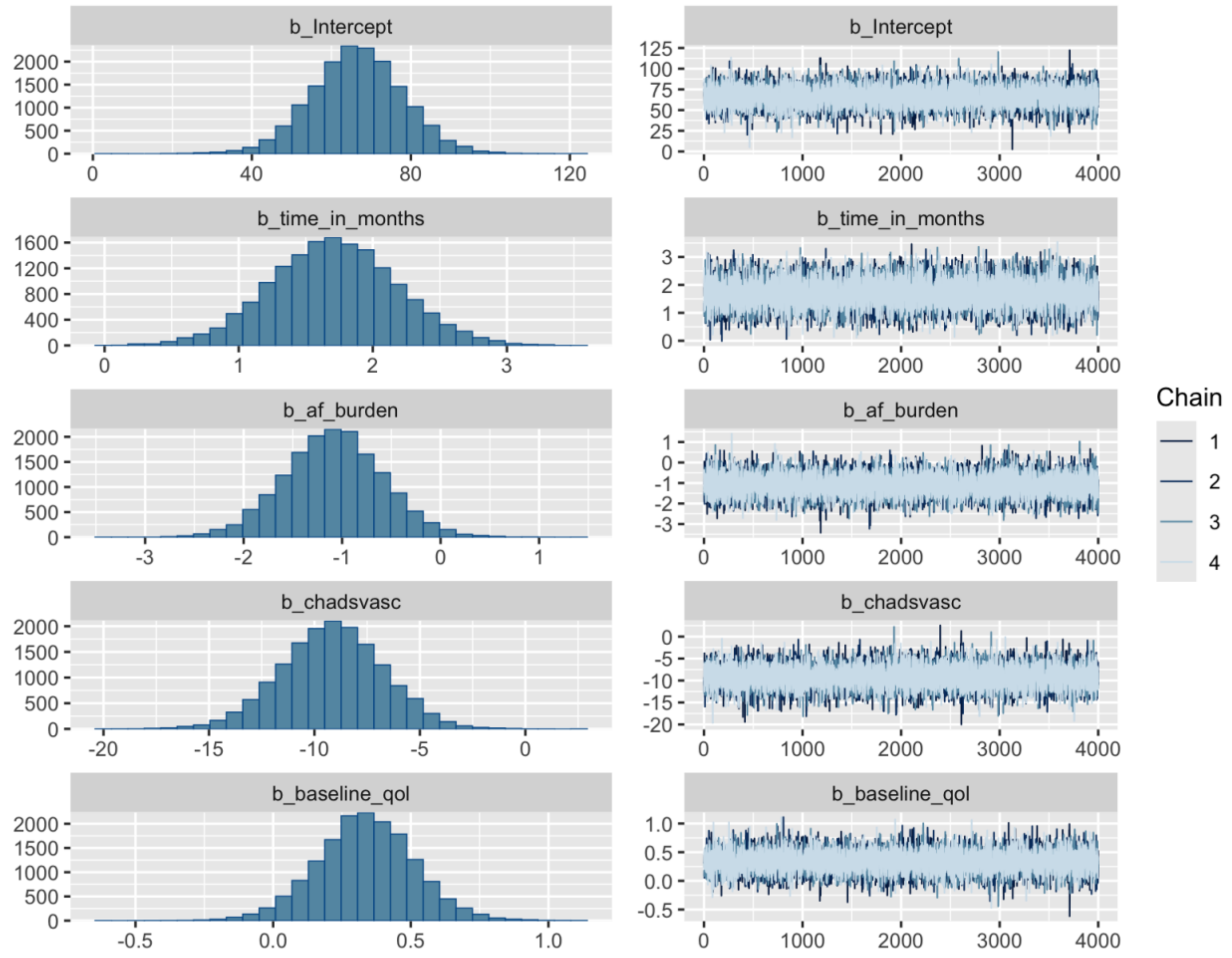

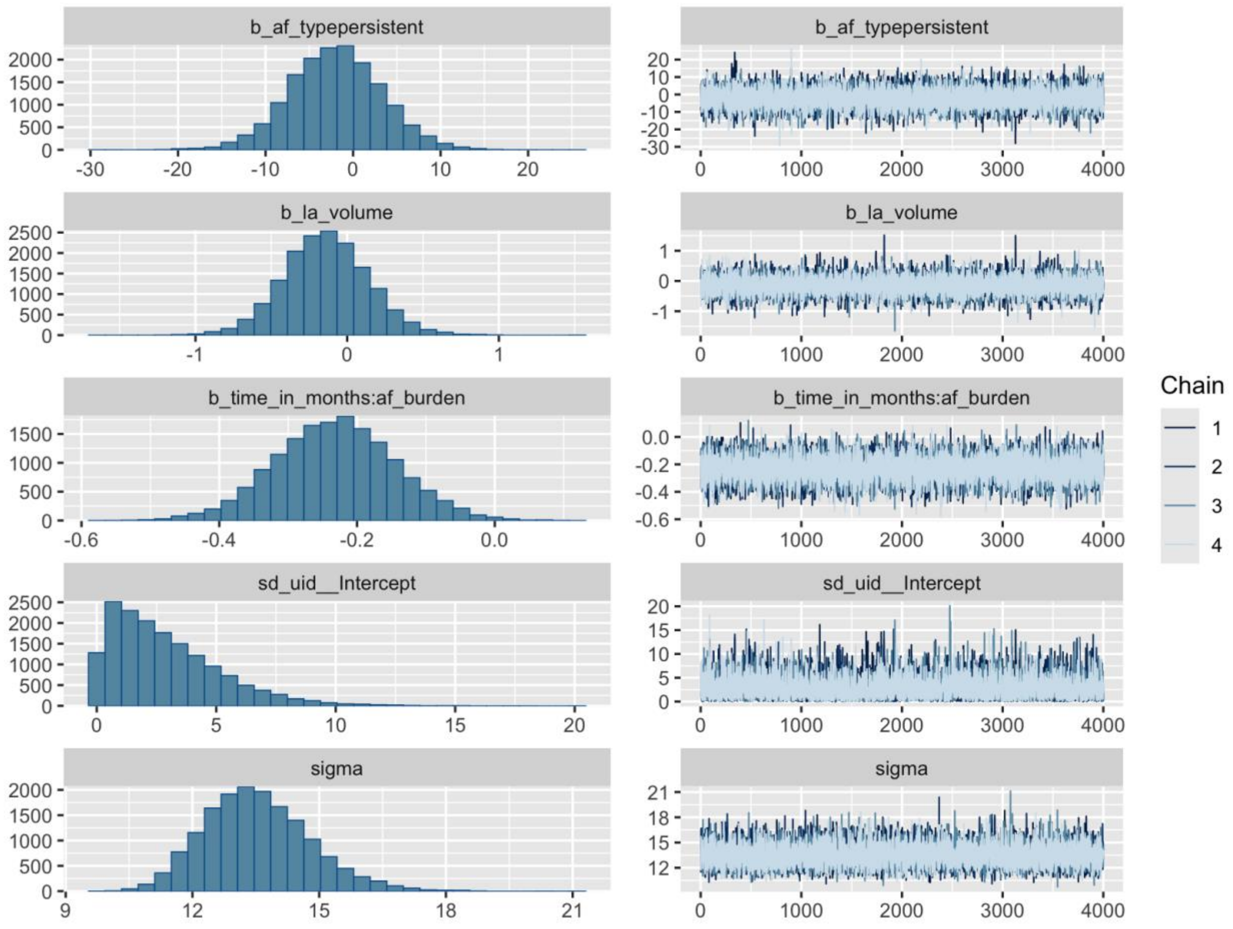
b_af_typepersistent
b_la_volume
b_time_in_months:af_burden
sd_uid__Intercept
sigma
Chain
1
2
3
4

Supplementary Figure 4. Trace and posterior density plots for binary recurrence model. Each row shows the posterior distribution (left) and trace plot across MCMC iterations (right) for the respective model parameter. Good chain mixing and overlapping density curves support adequate convergence.

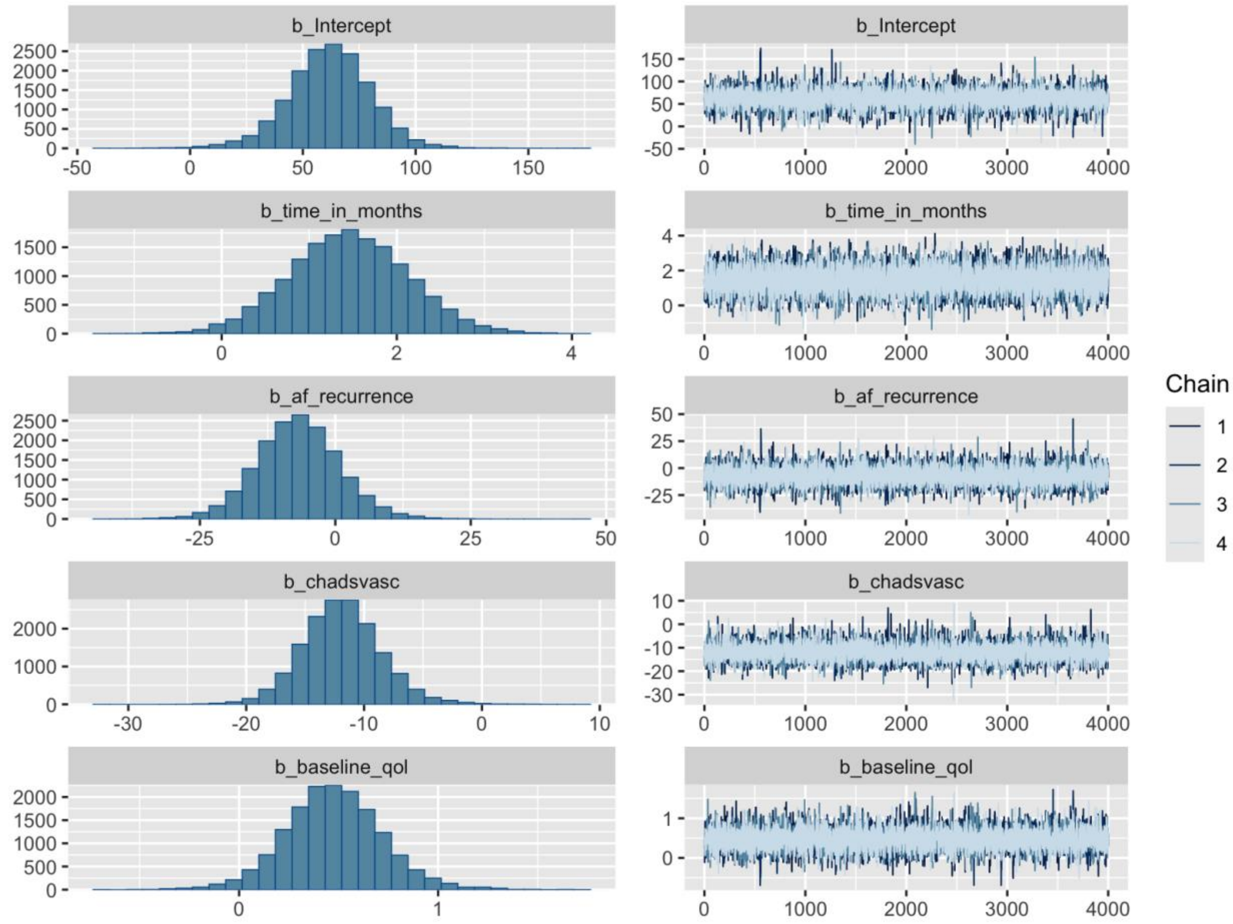

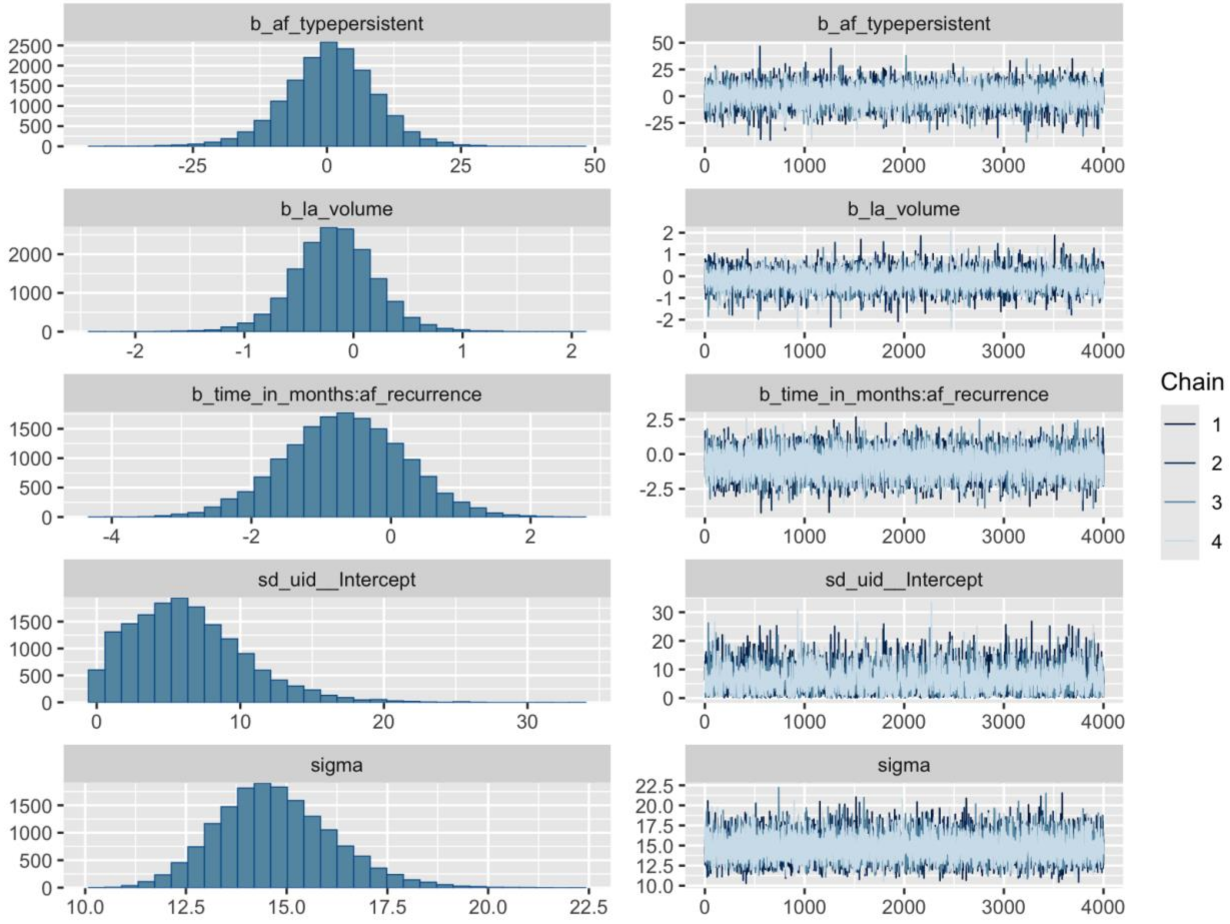
b_af_typepersistent
b_af_typepersistent
b_la_volume
b_la_volume
b_time_in_months:af_recurrence
b_time_in_months:af_recurrence
sd_uid__Intercept
sd_uid__Intercept
sigma
sigma
Chain
1
2
3
4

Supplementary Table 1. Convergence diagnostics for the continuous burden model. Reported are the $\hat{R}$ statistics (values close to 1 indicate convergence), and the effective sample sizes for bulk and tail estimates (ESS_bulk and ESS_tail).

variable	mean	sd	rhat	ess_bulk	ess_tail
b_Intercept	66.5044538	11.56574966	1.0001029	16,582.854	13,405.06
b_time_in_months	1.7117677	0.47371251	0.9999392	12,754.489	11,920.63
b_af_burden	-1.0871090	0.50120478	1.0002196	13,035.169	11,552.44
b_chadsvasc	-9.1630000	2.43628434	1.0002679	12,742.786	10,772.60
b_baseline_qol	0.3358116	0.17637727	1.0000717	12,324.118	11,493.78
b_af_typepersistent	-1.9952933	5.28181875	1.0004770	12,922.002	10,037.86
b_la_volume	-0.1391285	0.28150708	1.0004209	11,057.941	9,535.38
b_time_in_months:af_burden	-0.2316198	0.08720809	0.9999623	11,018.488	11,298.53
sd_uid__Intercept	2.8828848	2.29499060	1.0003894	5,434.598	7,557.65
sigma	13.5305134	1.24592362	1.0001252	16,118.579	11,459.05

Supplementary Table 2. Convergence diagnostics for the binary model. Reported are the $\hat{R}$ *statistics (values close to 1 indicate convergence), and the effective sample sizes for bulk and tail estimates (ESS_bulk and ESS_tail).*

variable	mean	sd	rhat	ess_bulk	ess_tail
b_Intercept	61.8356381	18.5290221	1.000663	10,059.760	8,839.328
b_time_in_months	1.4527028	0.6810861	1.000033	11,552.482	11,895.641
b_af_recurrence	-6.8539316	7.7578517	1.000078	9,757.747	8,791.268
b_chadsvasc	-11.8941162	3.3867748	1.000659	9,371.971	8,145.079
b_baseline_qol	0.4756410	0.2452050	1.000454	9,274.797	7,809.659
b_af_typepersistent	0.5870053	8.3991356	1.000772	10,106.780	8,947.157
b_la_volume	-0.1704043	0.3815924	1.000338	9,724.658	8,487.471
b_time_in_months:af_recurrence	-0.6651511	0.8599791	1.000046	11,576.470	10,786.859
sd_uid__Intercept	6.3394295	4.0044439	1.000769	3,090.308	6,062.245
sigma	14.7197618	1.4412948	1.000166	10,521.420	11,512.111

Supplementary Figure 5. Posterior predictive distribution overlay for the model with continuous AF burden. The observed data distribution (black line) is compared against simulated datasets (colored lines), indicating good model fit.

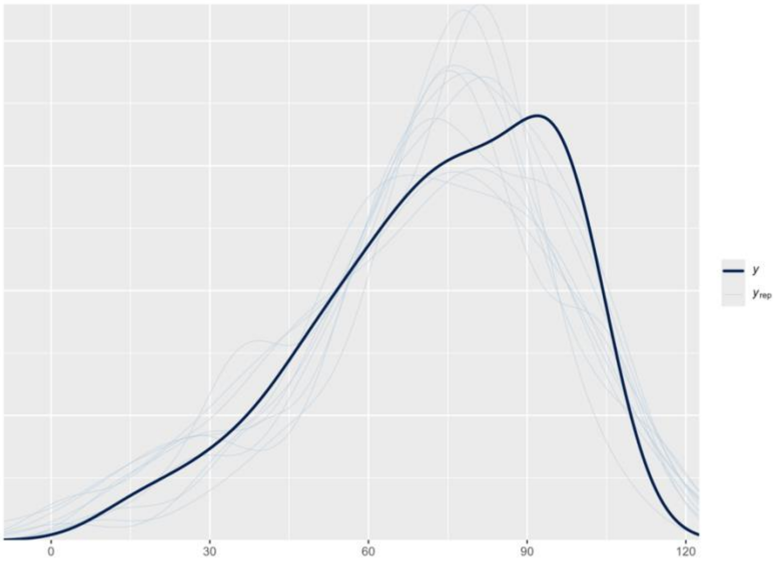

Supplementary Figure 6. Posterior predictive distribution overlay for the model with continuous AF burden. The observed data distribution (black line) is compared against simulated datasets (colored lines), indicating good model fit.

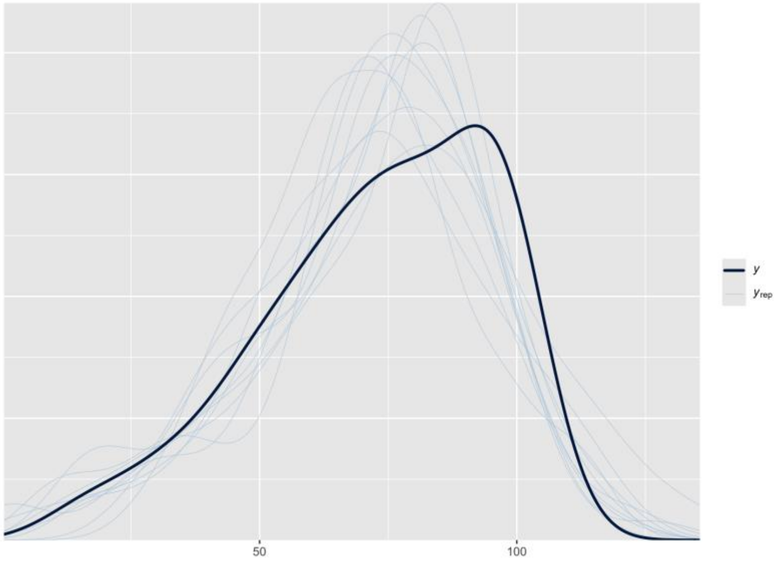

Bayesian model results:

Complete parameter estimates for the multivariable continuous and binary models are presented in Supplementary Tables 3 and 4, respectively. Both models adjusted for CHA_2DS_2-VASc score, baseline AFEQT, AF type, and indexed left atrial volume.

Supplementary Table 3. Parameter estimates for the model with AF burden.

Parameter	Estimate	95% Credible Interval
Intercept	66.50	43.89 to 89.27
Time (months)	1.71	0.78 to 2.65
AF burden (%)	-1.09	-2.07 to -0.1
CHA_2DS_2-VASc score	-9.16	-13.97 to -4.46
Baseline AFEQT	0.34	-0.01 to 0.69
AF type (persistent)	-2.00	-12.3 to 8.44
la_volume	-0.14	-0.68 to 0.41
Time × AF burden	-0.23	-0.4 to -0.06

Supplementary Table 4. Parameter estimates for the model with AF binary recurrence.

Parameter	Estimate	95% Credible Interval
Intercept	61.84	23.84 to 97.18

Parameter	Estimate	95% Credible Interval
Time (months)	1.45	0.13 to 2.79
af_recurrence	-6.85	-21.94 to 8.63
CHA_2DS_2-VASc score	-11.89	-18.26 to -4.92
Baseline AFEQT	0.48	0 to 0.97
AF type (persistent)	0.59	-16.79 to 16.66
la_volume	-0.17	-0.94 to 0.59
time_in_months:af_recurrence	-0.67	-2.36 to 1
